# Healthcare professionals' perceptions of pain in infants at risk for neurological impairment

**DOI:** 10.1186/1471-2431-4-23

**Published:** 2004-11-12

**Authors:** Lynn M Breau, Patrick J McGrath, Bonnie Stevens, Joseph Beyene, Carol S Camfield, G Allen Finley, Linda Franck, Alexandra Howlett, Karel O'Brien, Arne Ohlsson

**Affiliations:** 1 Pediatric Pain Service, IWK Health Centre, 5850 University Ave., P.O. Box 9700, Halifax, Nova Scotia, B3K 6R8, Canada; 2 Department of Psychology, Dalhousie University, Life Sciences Centre, Halifax, Nova Scotia, B3H 4J1, Canada; 3 Department of Pediatrics, Dalhousie University, 5850 University Ave., P.O. Box 9700, Halifax, Nova Scotia, B3K 6R8, Canada; 4 Faculty of Nursing, University of Toronto, 50 St. George St., Toronto, Ontario, M5S 3H4, Canada; 5 The Hospital for Sick Children Centre for Nursing, Room 4734B Atrium Building, 555 University Avenue Toronto, Ontario, M5G 1X8, Canada; 6 Population Health Sciences, The Hospital for Sick Children, 123 Edward Street, Room 407, Toronto, Ontario, Canada; 7 Department of Pediatrics, Department of Paediatrics, Mt. Sinai Hospital, 600 University Avenue, Toronto, Ontario, M5G 1X5, Canada; 8 Division of Neurology, IWK Health Centre, 5850 University Ave., P.O. Box 9700, Halifax, Nova Scotia, B3K 6R8, Canada; 9 Department of Anesthesiology, Dalhousie University, 5850 University Ave., P.O. Box 9700, Halifax, Nova Scotia, B3K 6R8, Canada; 10 Centre for Nursing and Allied Health Professionals Research, Great Ormond Street Hospital for Children NHS Trust, Great Ormond Street, London WC1N 3JH, UK

## Abstract

**Background:**

To determine whether healthcare professionals perceive the pain of infants differently due to their understanding of that infant's level of risk for neurological impairment.

**Method:**

Neonatal Intensive Care Units (NICU's) at two tertiary pediatric centers. Ninety-five healthcare professionals who practice in the NICU (50 nurses, 19 physicians, 17 respiratory therapists, 9 other) participated. They rated the pain (0–10 scale and 0–6 Faces Pain Scale), distress (0–10), effectiveness of cuddling to relieve pain (0–10) and time to calm without intervention (seconds) for nine video clips of neonates receiving a heel stick. Prior to each rating, they were provided with descriptions that suggested the infant had mild, moderate or severe risk for neurological impairment. Ratings were examined as a function of the level of risk described.

**Results:**

Professionals' ratings of pain, distress, and time to calm did not vary significantly with level of risk, but ratings of the effectiveness of cuddling were significantly lower as risk increased [F (2,93) = 4.4, p = .02]. No differences in ratings were found due to participants' age, gender or site of study. Physicians' ratings were significantly lower than nurses' across ratings.

**Conclusion:**

Professionals provided with visual information regarding an infants' pain during a procedure did not display the belief that infants' level of risk for neurological impairment affected their pain experience. Professionals' estimates of the effectiveness of a nonpharmacological intervention did differ due to level of risk.

## Background

Research on pain in infants has progressed considerably over the past twenty years. The nature and frequency of procedural pain in the neonatal intensive care unit (NICU) is now understood [1], many measures have been developed for assessment of acute pain in the NICU [2] and many pain interventions have now been evaluated [3].

However, much less is known about the pain experienced by neonates who are at risk for neurological impairment (NI), as most studies of neonatal pain have either excluded this group or have not examined data specific to them within larger data sets. We do know that this group represents approximately 10% of infants admitted to the Neonatal Intensive Care Unit [4] and that they experience more painful procedures in the NICU during the first days of life than infants who are not at risk for NI [5]. It also appears this vulnerable group may be particularly susceptible to potential long-term negative consequences of pain because of their neurological fragility, concomitant illnesses, and repeated exposure to painful stimuli [6].

The crucial first step of pain management is pain assessment. Without a valid and reliable approach to assessing pain, and the demonstrated efficacy of interventions for pain, decisions about pain management may not improve care. However, even with valid, reliable pain assessment tools, the characteristics of healthcare providers may affect ratings provided by them. These characteristics can include the healthcare providers' views of pain interventions [7], lack of awareness of advances in pain management [8], and use of pain cues that are not reliable [9].

The present study was designed to move beyond self-report of beliefs to examine whether healthcare professionals' judgments of pain in neonates are affected by their perception that a neonate has mild, moderate or severe risk for neurological impairment.

Research in this area is only emerging, but has important implications for how healthcare professionals deliver care to this vulnerable population. In a previous study using questionnaires, we found that caregivers of children with severe cognitive impairment view the pain of children with more severe impairment as reduced [10]. A second study using the same questionnaire with healthcare professionals and students revealed a similar pattern of beliefs [11]. Most recently, we adapted that questionnaire to assess the beliefs of healthcare professionals' regarding the pain of infants with varying degrees of risk for neurological impairment and again found that those who took part believed that the degree of pain experienced decreases as risk for neurological impairment increases [12].

These studies suggest that those who manage the pain of infants at risk for, or children with, intellectual deficits believe that pain is less for those at greater risk or who have greater impairment. These results may explain why we also found infants at risk for neurological impairment receive less pain treatment in the NICU [13]. However, to extrapolate from questionnaires to clinical behaviour can be problematic. Thus, the current study was designed as a step to linking these two sets of results. Specifically, we felt it was important to know if professionals' beliefs about pain in this group influence their assessment of infants' pain, which could lead to those infants' being provided with less pain treatment. As with any experimental study, the circumstances could not completely replicate those in a clinical setting. For example, the participants would not have access to physiological data or to the infants' recent history of pain. However, we hypothesized that the participants in this study would rate the pain lower for infants described as having greater risk for neurological impairment, corresponding with the beliefs expressed in our three previous studies.

## Methods

### Participants

One hundred and one healthcare professionals, with at least one year of experience working with infants with neurological impairment in the NICU, were recruited from two tertiary level university affiliated NICU settings in central and eastern Canada. They were recruited through information provided by the research nurses and notices posted within their centers. Each participant was paid a small honorarium for their participation, and all provided informed consent. The study was approved by each health centre's Research Ethics Board.

## Materials

### Demographic information

Participants completed a questionnaire that requested information regarding their age, gender, education and work experience.

### Video clips

Nine video clips were viewed by each participant. The 30 second video clips depicted term and preterm neonates of Caucasian descent experiencing a heel stick and squeezing for blood collection. Video clips were of the infants' faces only, with most lying on their sides and all bundled. Audio was not included. Prior to each videoclip, a verbal description of the neonate suggesting he/she was at mild, moderate or severe risk for neurological impairment was provided to the participant. These descriptions had been previously recorded on audio tape by a researcher to ensure each participant was read the description in an identical fashion. Descriptions were counterbalanced such that each videoclip was described for some participants as having either mild risk, moderate risk, or severe risk within each of two orders of presentation. Thus, each participant viewed three infants that were described as having mild, moderate or severe risk for neurological impairment, but the level of risk, and the order in which the clips were presented varied. Examples of the descriptions provided to participants are shown in Table 1.

**Table 1:** Sample descriptions of infants viewed on videotape provided

Mild Risk	Moderate Risk	Severe Risk
Brianna is 6 days old and has been treated for neonatal jaundice. She will make a complete recovery. Otherwise she is healthy.	Samuel was born 4 weeks prematurely and was mildly asphyxiated at birth because the cord was wrapped around his neck during delivery. An MRI shows a small area of damage in the brain.	Matthew was born with a serious metabolic condition which caused significant brain damage. He will likely not survive past 2 years of age.
Jason was born prematurely and is gaining weight slowly. He is now one month old. He suffered a unilateral Grade I bleed in his brain and will likely have no permanent damage from that.	Matthew was born with a serious metabolic condition which caused moderate brain damage. With the aid of a special diet, he will develop fairly well but will likely have significant learning disabilities.	Samuel was born 4 weeks prematurely and was severely asphyxiated at birth because the cord was wrapped tightly around his neck at delivery. An MRI shows extensive damage throughout the brain.

### Ratings of pain and distress

Participants rated the pain of infants shown on videotape from 0 (no pain) to 10 (extreme pain). They also rated each infant's pain using a Faces Pain Scale (0 = no pain, 6 = extreme pain). These measures were chosen because they are easy to use and were feasible for this experimental task. Although many validated measures of neonatal pain exist, these are multidimensional in nature. As such, they require the person using them to have access to information regarding the infant's physiological status, something we were unable to provide in the context of this task.

The 7 face scale [14], was included to allow a check of the validity of the 0–10 pain rating, since the latter is not commonly used in clinical neonatal settings. The Faces Pain Scale is also not typically used in a neonatal setting, but research indicates most adults find it easy to use [15], making it a useful check of participants' 0–10 pain ratings. Preliminary analyses indicated there was a significant relationship, similar to results for reliability computed for other sets of observational pain tools used in pediatric research [16], between 0–10 pain ratings and Faces Pain Scale ratings for infants at mild (r = .74), moderate (.r = .56) and severe risk for neurological impairment (r = .60).

### Ratings of cuddling and calm

Participants provided a rating of how effective they believed a behavioural intervention (i.e. cuddling) would be for minimizing the procedural pain for each infant (0 = no effect, 10 = very effective) and of how long (seconds) they believed it would take each infant to calm without intervention.

### Ratings of risk for neurological impairment

To ensure that the descriptions provided were valid depictions of infants at each level of risk and were understood and accepted by participants, participants were asked to rate the level of risk they believed each infant had for future neurological impairment (0 = no risk, 10 = certain impairment).

### Procedure

Participants took part in small groups of 5 to 6 professionals that were randomly assigned to one of the two orders of presentation. They completed the demographic questionnaire and the rating tasks were explained to them. They were then shown the nine video clips. After each videoclip was viewed, the participants were provided with time to make their independent ratings for that videoclip before the next was shown. Participants were not permitted to interact with each other until all tapes had been viewed and rated. After ratings were complete, they were debriefed regarding the purpose of the study and a discussion of their experience was facilitated by the research assistant.

### Preliminary analyses

#### Exclusions due to missing data

Preliminary analyses indicated that two participants were missing more than 10% of ratings. As per an a priori decision as to how to handle missing data, their data were excluded from further analyses. The remaining 99 participants were missing 0% (n = 88) to 7% (n = 1) of responses (M = 0.2, SD = 0.6).

#### Exclusions due to presentation order effects

A 2 (group) X 6 (rating) Repeated Measures Analysis of Variance (RM ANOVA) was conducted on the six ratings provided by participants who viewed the tapes in the two orders to determine whether order of presentation had affected ratings. This analysis revealed a significant effect of order of presentation [F(1,97) = 4.4, p = .04). A more detailed look at the data using stem and leaf plots revealed 4 participants in one group had extreme scores for ratings of Time to Calm (M = 192.8, SD = 39.2) relative to the other participants in that group (M = 37.4, SD = 28.7). The data of these four participants were removed. A second RM ANOVA revealed no significant effect due order of presentation of the video clips [F(1,93) = 1.7, p = .20). Thus, 95 professionals formed the final sample for the study.

#### Manipulation check

To determine if the descriptions provided to the participants were effective in leading them to believe the infants viewed had mild, moderate or severe risk for neurological impairment, each participant's mean rating for degree of risk for future impairment for the infants they were told had a mild (3), moderate (3) or severe risk (3) for impairment were computed. These ratings were then compared using a RM ANOVA. There was a significant difference in the ratings provided to those clips described as having mild (M = 3.8, SD = 2.8), moderate (M = 4.9, SD = 2.0) and severe risk (M = 6.1, SD = 2.8; F(2, 93) = 13.9, p < .001). Post-hoc paired t-tests indicated these differences were significant between infants described as having mild and moderate risk (t((94) = -3.2, p = .002), mild and severe risk (t((94) = -4.8, p < .001), and moderate and severe risk (t((94) = -5.0, p < .001), Thus, participants believed the infants had different levels of risk when they provided ratings for the video clips.

### Statistical procedures

The data were analyzed using SPSS Version 10.0.7 [17]. Power computations were completed using Sample Power 1.2 [18] or based on tables prepared by Stevens [19]. Alpha was set at .05 for all tests and Bonferroni corrections were applied to sets of post hoc matched sample t-tests to maintain alpha at .05 for each set. Because the corrected p values varied with the number of tests in each set, raw p values are reported. Wilks Lambda was used to test significance for all RM ANOVA's. There was .80 power or greater to detect medium size effects using repeated measures analyses with 3 to 5 levels of factors and greater than .99 power to detect medium size differences in means using matched sample t-tests. Power was .86 to detect a significant medium size correlation between ratings and years of experience.

#### Descriptive statistics

Descriptive statistics were generated for the demographic characteristics of the participants (Table 2) and for the ratings provided for each set of video clips (Table 3).

**Table 2:** Characteristics of the participants (N = 95)

Characteristic	n	%
Site		
Toronto	46	48
Halifax	49	52
Profession		
Nurse	50	53
Physician	19	20
Respiratory Therapist	17	18
Occupational Therapist	2	2
Physiotherapist	2	2
Psychologist	1	1
Other Clinician	4	4
Gender		
Female	82	86
Age		
20–30 years	19	20
31–35 years	15	16
36–40 years	32	34
41–45 years	11	12
46 years or more	18	19

Note. Percentages rounded.

**Table 3:** Mean ratings given to infants described as at risk for mild, moderate or severe risk for neurological impairment (N = 95)

Rating	Mild Risk		Moderate Risk		Severe Risk	
	M	SD	M	SD	M	SD

Pain (0–10)	6.3	1.7	6.6	1.7	6.2	1.6
Faces pain Scale (0–6)	4.7	1.0	4.8	0.8	4.8	1.0
Distress (0–10)	6.1	1.7	6.5	1.5	6.3	1.6
Effectiveness of Cuddling (0–10)	7.0	2.1	7.1	1.9	6.5	2.2
Time to calm (Seconds)	35.2	34.8	34.9	28.3	32.8	31.8

#### Effect of risk for neurological impairment on ratings

To compare the ratings provided for the 9 video clips, a 3 (level of risk) X 5 (rating type) RM ANOVA was conducted on the scores of the 95 participants. This was followed by 5 one-way RM ANOVA's on each rating (0–10 pain rating, Faces Pain Scale rating, distress rating, effectiveness of cuddling rating, time to calm) and matched sample t-tests on ratings when the one-way ANOVA was significant.

#### Effect of participants' characteristics on ratings

The effect of participants' characteristics on ratings was examined using Mixed Measures ANOVA's on the five ratings at three levels of risk. The first three included Gender, Age, and Site (Toronto, Halifax) as between-subjects effects. The fourth included three levels of profession (i.e. staff nurse, physician, respiratory therapist) as the between-subjects effect. Other professionals were not included due to small numbers. The relation between the participants' years of experience in a neonatal setting and their ratings were investigated using Pearson Correlations.

## Results

### Participants

The characteristics of the participants are displayed in Table 2. The majority were nurses and the number of years experience in a neonatal setting ranged from 1.5 to 36 years (M = 11.8, SD = 7.7). The 50 nurses included staff nurses (n = 34), advanced practice nurses (n = 9) and nurse managers/educators (n = 7). The physicians specialized in neonatology (n = 10), neurology (n = 4), pediatrics (n = 3) and other specialties (n = 2). Six of the 19 physician participants were residents or fellows. Additional professions are listed in Table 2.

### Effect of risk for neurological impairment on ratings

The mean ratings provided for video clips of infants described as having mild, moderate or severe risk for neurological impairment are depicted in Table 3. The RM ANOVA on the five ratings revealed a nonsignificant effect of Level of Risk [F (2,93) = 0.6, p > .05], a significant effect of Rating [F (4,91) = 91.6, p < .001] and a significant interaction between the two [F (8,87) = 3.4 p = .002]. Thus, there was no overall effect due to the level of risk described, but level of risk described did affect some ratings.

One-way RM ANOVA's revealed Level of Risk had a marginal effect on participants' ratings of pain on the 0–10 scale [F (2,93) = 2.9, p = .06] and a significant effect on ratings of the perceived effectiveness of cuddling [F (2,93) = 4.4, p = .02], but nonsignificant effects on Faces Pain Scale ratings [F (2,93) = 0.3, p = .70], distress [F (2,93) = 2.2, p = .12] and time to calm [F (2,93) = 0.4, p = .65]. As Table 3 shows, there was a slight tendency for participants to rate pain lower for infants who were described as having greater risk for impairment. Participants did believe cuddling would be less effective when risk for neurological impairment was greater. Ratings of the effectiveness of cuddling were significantly lower for infants described as at high risk than they were for those described as at mild risk [t(94) = 2.5, p = .01] or moderate risk [t(94) = 3.0, p = .004]. The difference in ratings between those described as at mild or moderate risk were nonsignificant [t(95) = -0.2, p = .77]. Thus, participants believed that beyond a moderate level of risk, the effectiveness of cuddling dropped significantly.

In summary, participants did not view the pain of the infants as varying due to level of risk for neurological impairment. Nor did they perceive the distress or time to calm after pain as differing between groups of infants described as having mild, moderate or severe risk for neurological impairment. However, they did perceive that cuddling would be less effective as an intervention for infants with high risk, than for those with mild or moderate risk of neurological impairment.

### Effect of participants' characteristics

#### Site, gender and age

Three Mixed Measures ANOVA's were used to examine the effect of participants' characteristics on the five ratings ratings. The first result indicates a nonsignificant main effect of Site [F(1,93) = 0.7, p = .39], the second revealed a nonsignificant main effect of Gender [F(1,93) = 0.9, p = .34], and the third indicated Age also did not significantly effect ratings on the five measures [F(4,90) = 0.2, p = .95]. Thus, participants' ratings did not vary due to their institution, gender or age.

#### Profession

To examine the effect of participants' profession on their ratings, three groups were included in a Mixed Measures ANOVA: staff nurses (n = 34), physicians (n = 11), and respiratory therapists (n = 17). Residents and Fellows, Nurse Managers and Educators and Specialists, and other professionals were not included due to small numbers in those groups. The analysis revealed a significant main effect of Rating Scale [F(4,56) = 3.9, p = .001]. However, the main effect of Level of Risk was nonsignificant and the main effect of Profession only approached significance [F(2,59) = 2.7, p = .07]. Participants' ratings were not affected by their professional background. The interaction between Rating and Level of Risk was significant [F(8,52) = 36.6, p < .001], but all other interactions were nonsignificant. Games-Howell post-hoc comparisons revealed a significant difference in the ratings provided by staff nurses and physicians (p = .004) and a difference between respiratory therapists and physicians that approached significance (p = .06). As shown in Table 4, Nurses' ratings did not appear to differ greatly due to level of risk for impairment, while Physicians' showed a tendency to rate all aspects of the experience higher as level of risk increased, and respiratory therapists tended to provide lower ratings as the infants' level of described risk for neurological impairment increased.

**Table 4:** Mean pain, distress, effectiveness of cuddling and time to calm scores given to infants described as at risk for mild, moderate or severe risk for neurological impairment by physicians and other clinicians

Rating	Level of Risk	Staff Nurses (n = 34)		Physicians (n = 11)		Respiratory Therapists (n = 17)	
		M	SD	M	SD	M	SD

0 – 10 Pain rating	Mild	6.4	1.6	4.8	2.3	6.9	1.3
	Moderate	6.9	1.5	5.6	2.4	6.9	1.8
	Severe	6.4	1.7	5.8	2.1	5.8	1.6
Faces pain rating (0–6)	Mild	4.6	1.0	3.7	0.8	5.3	0.6
	Moderate	4.9	0.7	4.8	0.6	4.9	1.1
	Severe	4.9	1.0	5.4	0.6	4.3	1.3
0 – 10 Distress rating	Mild	6.4	1.6	4.3	2.1	6.9	1.3
	Moderate	6.8	1.3	5.6	2.2	6.8	1.9
	Severe	6.4	1.6	6.3	1.9	5.4	1.6
0–10 Effectiveness of cuddling rating	Mild	6.4	1.6	4.3	2.1	6.9	1.3
	Moderate	7.2	2.0	6.9	1.9	6.5	2.4
	Severe	6.6	2.3	5.8	2.6	4.9	2.1
Time to calm estimate (seconds)	Mild	40.0	36.1	14.9	15.5	46.8	43.8
	Moderate	39.8	28.9	18.4	13.8	42.2	35.4
	Severe	40.5	40.3	23.3	15.7	31.3	33.0

#### Professional experience

Eighty-nine participants provided information regarding their amount of professional experience. Correlations indicated that years of experience were not correlated significantly with any of the five ratings provided after corrections for multiple tests. Thus, the importance of an infants' level of risk for neurological impairment was neither greater nor less as experience in this setting increased.

## Discussion

Overall, the professionals in this study did not rate the pain of neonates differently when provided with information indicating those infants had mild, moderate or severe risk for neurological impairment. The professionals' perception of the infants' level of risk also did not affect their ratings of the infants' distress, or their belief in how long the infant would take to calm after pain without intervention. Professionals did perceive that cuddling would be significantly less effective for infants at high risk for neurological impairment than for infants with mild or moderate impairment. However, this effect was not large, and, although it was statistically significant, it may be spurious. Further research should examine whether beliefs regarding pain experience in this group and beliefs regarding the effectiveness of cuddling and other nonpharmacological interventions are truly independent. These results are inconsistent with the results of our previous questionnaire study indicating professionals, with similar levels of experience in neonatal intensive care settings, perceive the pain experience of infants as reduced as their level of risk for neurological impairment increases [12]. There are several possible reasons for these discrepant results.

The professionals who participated in this study were asked to rate the risk for neurological impairment of each infant they viewed on videotape. Asking them to do this may have alerted them to the purpose of the study and elicited efforts on their behalf to provide ratings that were unbiased. However, their ratings of the perceived effectiveness of cuddling did vary by level of risk for impairment, suggesting attempts to appear unbiased do not fully explain the results found.

In our previous studies, questionnaires elicited beliefs about the pain experience of infants and children with varying levels of risk *relative to *the pain experience of those without risk [10-12]. In contrast, no infants in the current study were described as having no risk for neurological impairment. This was because the infants' appearance made it apparent that they were not healthy full-term infants. It may be that the comparative nature of the questions in the previous studies made the possibility of differences in pain experience due to neurological risk more salient to participants. Thus, the pain ratings provided here did not differ among levels of risk, but had ratings of healthy infants been included in the task, they may have differed significantly from them.

It is also possible that the beliefs expressed by professionals in our previous study [12] do not moderate professionals' behaviour in relation to pain assessment for specific infants, as was found here. A discordance between expressed beliefs and behaviour, in regard to pediatric pain management, has been reported elsewhere [20,21]. Thus, the professionals here may hold similar beliefs to the professionals in our previous study, but these beliefs did not alter their behaviour when asked to judge pain in a specific infant based on observable behaviour. This interpretation is supported by the current results because no differences were found due to level of risk for ratings that the professionals could base on behaviour they observed on the video clips: pain, distress, time to calm. In contrast, professionals' judgments of the effectiveness of cuddling were influenced by the descriptions of the infants' level of risk for neurological impairment. This may be because there was no visual information to base this rating upon, so professionals used the descriptions of risk provided, presumably in light of their previous experience with these groups in the neonatal setting.

The finding that pain ratings did not vary due to level of risk for neurological impairment raises questions about our previous study that revealed infants at risk for neurological impairment receive less pain treatment in the NICU [13]. When a group is provided less medication for pain, it is typically assumed that this is because their pain was judged as less. However, it is possible that professionals hold beliefs about pain treatment that directly impact upon treatment decisions, irregardless of pain assessment. For example, they may hold beliefs about the appropriateness of medication for specific groups that are unrelated to beliefs about the amount of pain that group experiences. In support of this perspective, research indicates that nurses hold negative attitudes towards pharmacological treatment for pain [7] and that steps to improve pain assessment do not necessarily result in changes in pain management [22].

Further research is needed to reconcile the current results with beliefs that risk for neurological impairment does affect pain experience expressed by a similar group of professionals in our previous survey [12] and the results of our study indicating procedural pain is not treated as frequently for infants in the NICU who have greater risk for neurological impairment [13]. If this reflects a disconnect between pain beliefs related to assessment and those related to treatment for infants at risk for neurological impairment, then educational interventions aimed at improving care through changes in pain assessment may be ineffective. In that case, other avenues to changing professionals' pain management for this group should be explored.

Another finding in this study warrants discussion. Professionals' judgments of the effectiveness of cuddling decreased with increasing risk for neurological impairment, despite their having judged pain as similar in intensity. This result is similar to a finding by Fanurik et al. [23]. They found nurses, but not physicians, responding to vignettes of children undergoing painful procedures, indicated nonpharmacological interventions would be less appropriate as level of cognitive impairment increased. The same professionals' ratings of the pain intensity experienced by the children in that study did not differ due to perceived level of cognitive impairment.

The current results, along with those of Fanurik's group [23], raise the question of whether professionals perceive the pain experienced by those at risk for or with neurological impairment as similar in intensity, but differing in quality from those at lesser risk. Because the current study elicited ratings only of the intensity of pain and distress and professionals were not asked about the nature of the pain the infants experienced, the results cannot confirm this possible explanation, as data regarding pain quality was not collected. However, professionals in our survey study differentiated between physiological aspects of pain and internal and external responses to pain, such as emotional reaction, behavioural reaction and communication of pain [12]. They also believed the experience of infants at greater risk was more reduced along the latter aspects that are more psychological in nature. Caregivers' have expressed similar beliefs, and also perceived the behaviour of children with more severe impairment is more closely related to their physiological pain experience [10]. From this finding, we could suggest that there is a belief, on the part of professionals and caregivers, that the pain behaviour of those at greater risk for, or with, neurological impairment is more reflexive in nature. We could further speculate that the underlying rationale may be that they are seen as less able to interpret their pain, both cognitively and emotionally, due to their neurological impairment. However, we would need to conduct further research to substantiate this rationale.

If professionals and caregivers do believe pain behaviour is more reflexive, and that pain experience is more physiologically based when a child has neurological impairment, it could explain the current results regarding the effectiveness of cuddling. Professionals viewing the video clips may have perceived the behavioural responses of the infants with different levels of risk for impairment as being similar in intensity. Nonetheless, they may have interpreted the behaviour of those with more risk as more of a reflexive response to a physiological insult, while they saw the behaviour of those with lesser risk as reflecting a more multidimensional pain experience incorporating both physical and psychological suffering. Thus, we could again speculate that they may have felt cuddling, an intervention that would address physical and psychological aspects of pain, would be more effective for the less impaired groups. This phenomenon would not be novel or unique. For most of recorded history, there has been a belief that cognitive interpretation of pain was necessary for pain to result in long-term negative consequences. This belief was often the justification for poorer pain management for both children and infants [24]. Although this belief is fading in regard to children and infants in general, it is still held in relation to those who are most severely at risk for, or have neurological impairment, and are perceived as least capable of interpreting their pain. Alternatively, this belief may be based on the actual experience of professionals in this study, that it is more difficult to calm an infant at risk for neurological impairment. This experience may also be an accurate perception of the difficulty infants at greater risk for impairment may have in responding to behavioural interventions because of their reduced ability to organize behavioural state and biobehavioural responses. Further research should examine these areas of speculation to specifically determine whether the perception that a behavioural intervention will be less effective for infants at greater risk for neurological impairment does reflect professionals' direct experience with this group or their understanding of how the pain experience may be affected by neurological impairment that may affect pain interpretation.

The current study has several limitations. Professionals were asked to rate the pain experience of infants receiving heel sticks from videotape. Although this may approximate the real situation in a NICU setting, it is not identical. In a NICU setting, professionals would have rich information from the environment, previous contact with an infant, physiological data, and medical records that guide their assessment of pain. They would also view this infant within the context of all other infants in the unit. Professionals here were also asked only to provide ratings of pain intensity. As the results suggest, this is only one dimension of pain and may not be the dimension that plays the largest role in their judgments regarding pain in a clinical setting. The professionals here were experienced in the types of pain experienced in the NICU and may have held a priori beliefs about the painfulness of this procedure that moderated their judgments. Research suggests professionals' beliefs regarding the painfulness of a procedure play a large role in their assessments of children's pain [7,9,25].

## Conclusions

The current study indicates professionals' perception of the pain intensity of infants does not differ due to their understanding of the infants' level of risk for neurological impairment. Professionals also view cuddling as less effective for infants at greater risk for neurological impairment. Further research is needed to examine the reasoning behind the judgments made by healthcare professionals and to clarify why they might view an intervention as less effective for infants with greater risk of neurological impairment, despite having rated their pain intensity as similar to that of infants at lesser risk.

## Competing interests

The authors declare that they have no competing interests.

## Authors' contributions

The study was conceived and designed by BS, PM & LB with assistance from all remaining authors. The study was conducted under the supervision of PM, BS, KO, AO. Statistical analyses were conducted by LB, with assistance from PM, BS and JB. Interpretation of results were conducted by LB, PM, BS, JB, CC, LF, KB and AO. The manuscript was prepared by LB, and edited by PM and BS, with review and assistance from all remaining authors. All authors read and approved the final manuscript.

## Pre-publication history

The pre-publication history for this paper can be accessed here:


